# Data on burden of comorbidities in the united states and medicaid expansion status

**DOI:** 10.1016/j.dib.2016.05.019

**Published:** 2016-05-20

**Authors:** Tomi Akinyemiju, Justin Xavier Moore

**Affiliations:** a Department of Epidemiology, University of Alabama at Birmingham, Birmingham, AL, United States; b Comprehensive Cancer Center, University of Alabama at Birmingham, Birmingham, AL, United States

**Keywords:** Comorbidities, Medicaid expansion, Affordable Care Act, Disparities

## Abstract

The high prevalence of comorbidities among US adults is a major public health problem. However, there is limited data on the geographic distribution of comorbidities. In addition, recent changes to health insurance programs in the US through the Affordable Care Act, and the Medicaid expansion program specifically, has the potential to significantly improve the prevention and management of comorbid conditions in the US. In a recent analysis, we examined disparities in the burden of comorbidities among US adults by state Medicaid expansion status, (Akinyemiju et al., 2016) [1]. Here, we provide additional data showing the state level mean number of comorbidities in all 50 US states for African–Americans and whites, stratified by Medicaid expansion status. In addition, we provide a map of the US states showing the geographic distribution of comorbidities and stratified by race/ethnicity and gender.

**Specifications Table**

**Table:** 

Subject area	*Epidemiology; Health Policy*
More specific subject area	*Descriptive epidemiology and disease prevention*
Type of data	*Table, figure*
How data was acquired	*Online from 2013 Behavioral Risk Factor Surveillance System*
Data format	*Analyzed*
Experimental factors	*Generalized linear model to estimate state-level mean comorbidities*
Experimental features	*Geospatial mapping of state-level mean comorbidities.*
Data source location	*50 US states –*www.cdc.gov
Data accessibility	*Data are within this article*

**Value of the data**

• This data provides detailed information on the burden of comorbid conditions among US adults residing in all 50 states. We include race and gender stratified estimates to better inform population-specific health programs
• As the roll-out and implementation of the Medicaid expansion program continues, this data may provide valuable information on baseline values to which future estimates may be compared
• US states that are considering Medicaid expansion may benefit from this data by identifying the health need of their population in terms of comorbidities, and understanding the potential impact on the health of the population, and the cost-effectiveness of improved healthcare access

## 1 Data

We performed cross-sectional analysis of socio-demographic, behavioral and risk factor data from the 2013 Behavioral Risk Factor Surveillance System (BRFSS) dataset [1], [2]. We accessed data for this study from the 2013 BRFSS data file available at http://www.cdc.gov/brfss/annual_data/annual_2013.html, and identified state expansion status using data from the Center for Medicare and Medicaid Services report on State Medicaid and CHIP Income Eligibility Standards, based on state decisions as of January 1, 2015 [3].

## 2 Experimental design, materials and methods

### 2.1 Burden of Comorbidities

Among 491,773 BRFSS survey participants; we excluded 7908 due to missing data, corresponding to a total of 483,865 participants. The overall age-adjusted mean number of comorbidities in all 50 states was 2.23 (95% CI: 2.22-2.24). The mean number of comorbidities ranged from 1.95 (95% CI: 1.92-1.99) in Hawaii to 2.52 (95% CI: 2.47-2.58) in Alabama.

### 2.2 Statistical analysis

We performed all analyses using SAS version 9.3. We applied appropriate statistical weights and strata to account for clustering and sampling design for BRFSS. We categorized each state as expanded or non-expanded based on adoption of the Affordable Care Act (ACA). To estimate the age-adjusted state-level mean comorbidities, we performed a generalized linear regression model with number of comorbidities as the outcome, state as the exposure, age as a covariate, and accounted for strata and statistical weights. We additionally stratified the state-level mean number of comorbidities by gender and race.
