# Supplementary material for: Data on burden of comorbidities in the united states and medicaid expansion status
Source: Data Brief. 2016 May 20;8:120–2. doi: 10.1016/j.dib.2016.05.019 (PMC4889874; doi:10.1016/j.dib.2016.05.019)
Supplement: Supplementary file 2 — Supplementary material [file mmc2.zip › Prevalence of Comorbidities Tables Figures JXM.docx]

| **Table 1: State-level mean number of comorbidities and Medicaid expansion status based on 2013 BRFSS.** | | | | |
| --- | --- | --- | --- | --- |
| State | N | Estimated N | Expanded | Age-Adjusted Mean Comorbidities |
| Alabama | 6503 | 477,569 | No | 2.52 (2.47 – 2.58) |
| Alaska | 4578 | 121,963 | No | 2.10 (2.04 – 2.16) |
| Arizona | 4252 | 673,366 | Yes | 2.24 (2.16 – 2.31) |
| Arkansas | 5268 | 421,995 | Yes | 2.42 (2.36 – 2.49) |
| California | 11518 | 3,618,219 | Yes | 2.09 (2.06 – 2.12) |
| Colorado | 13649 | 1,030,345 | Yes | 2.07 (2.04 – 2.09) |
| Connecticut | 7710 | 299,681 | Yes | 2.19 (2.16 – 2.23) |
| Delaware | 5206 | 95,830 | Yes | 2.26 (2.21 – 2.32) |
| District of Columbia | 4931 | 57,158 | Yes | 2.22 (2.16 – 2.28) |
| Florida | 34186 | 1,965,422 | No | 2.27 (2.23 – 2.30) |
| Georgia | 8138 | 1,024,810 | No | 2.31 (2.27 – 2.36) |
| Hawaii | 7858 | 198,402 | Yes | 1.95 (1.92 – 1.99) |
| Idaho | 5630 | 304,430 | No | 2.12 (2.06 – 2.17) |
| Illinois | 5608 | 1,902,719 | Yes | 2.12 (2.07 – 2.17) |
| Indiana | 10338 | 864,539 | No | 2.33 (2.28 – 2.37) |
| Iowa | 8157 | 566,085 | Yes | 2.17 (2.13 – 2.22) |
| Kansas | 23282 | 424,133 | No | 2.18 (2.16 – 2.20) |
| Kentucky | 11013 | 472,937 | Yes | 2.45 (2.41 – 2.49) |
| Louisiana | 5251 | 610,590 | No | 2.30 (2.23 – 2.38) |
| Maine | 8097 | 228,324 | No | 2.32 (2.27 – 2.36) |
| Maryland | 13011 | 669,566 | Yes | 2.18 (2.14 – 2.21) |
| Massachusetts | 15071 | 653,812 | Yes | 2.22 (2.18 – 2.25) |
| Michigan | 12759 | 1,317,205 | Yes | 2.40 (2.37 – 2.44) |
| Minnesota | 14340 | 758,400 | Yes | 2.04 (2.02 – 2.07) |
| Mississippi | 7453 | 489,198 | No | 2.40 (2.35 – 2.45) |
| Missouri | 7118 | 1,047,830 | No | 2.32 (2.27 – 2.37) |
| Montana | 9693 | 193,039 | No | 2.14 (2.11 – 2.18) |
| Nebraska | 17139 | 375,919 | No | 2.14 (2.11 – 2.17) |
| Nevada | 5101 | 357,824 | Yes | 2.17 (2.11 – 2.24) |
| New Hampshire | 6463 | 161,373 | Yes | 2.29 (2.25 – 2.33) |
| New Jersey | 13386 | 942,366 | Yes | 2.11 (2.08 – 2.14) |
| New Mexico | 9316 | 366,004 | Yes | 2.15 (2.11 – 2.19) |
| New York | 8979 | 2,110,228 | Yes | 2.18 (2.15 – 2.22) |
| North Carolina | 8860 | 1,570,209 | No | 2.33 (2.29 – 2.37) |
| North Dakota | 7806 | 121,766 | Yes | 2.10 (2.05 – 2.14) |
| Ohio | 11971 | 1,308,952 | Yes | 2.34 (2.30 – 2.37) |
| Oklahoma | 8244 | 658,870 | No | 2.38 (2.34 – 2.42) |
| Oregon | 5949 | 426,480 | Yes | 2.32 (2.28 – 2.37) |
| Pennsylvania | 11429 | 1,441,939 | Yes | 2.27 (2.24 – 2.31) |
| Rhode Island | 6531 | 113,452 | Yes | 2.37 (2.33 – 2.41) |
| South Carolina | 10717 | 780,734 | No | 2.39 (2.36 – 2.43) |
| South Dakota | 6895 | 122,535 | No | 2.08 (2.03 – 2.13) |
| Tennessee | 5815 | 629,698 | No | 2.41 (2.36 – 2.47) |
| Texas | 10917 | 3,942,233 | No | 2.18 (2.14 – 2.22) |
| Utah | 12769 | 509,957 | No | 2.17 (2.14 – 2.20) |
| Vermont | 6392 | 95,463 | Yes | 2.22 (2.19 – 2.26) |
| Virginia | 8464 | 940,040 | No | 2.23 (2.20 – 2.27) |
| Washington | 11162 | 600,889 | Yes | 2.26 (2.23 – 2.29) |
| West Virginia | 5899 | 395,710 | Yes | 2.58 (2.53 – 2.63) |
| Wisconsin | 6589 | 998,089 | No | 2.17 (2.12 – 2.22) |
| Wyoming | 6454 | 73,879 | No | 2.17 (2.11 – 2.23) |
| Total | 483,865 | 39,532,178 | - | 2.23 (2.225 – 2.242) |

| **Table 2: State-level mean number of comorbidities and Medicaid expansion status based on 2013 BRFSS.** Among White women alone. | | | | |
| --- | --- | --- | --- | --- |
| State | N | Estimated N | Expanded | Age-Adjusted Mean Comorbidities |
| Alabama | 3,051 | 193,107 | No | 2.60 (2.52 – 2.68) |
| Alaska | 1,796 | 40,919 | No | 2.17 (2.08 – 2.27) |
| Arizona | 1,870 | 257,969 | Yes | 2.35 (2.22 – 2.49) |
| Arkansas | 2,532 | 185,740 | Yes | 2.47 (2.37 – 2.56) |
| California | 3,758 | 893,816 | Yes | 2.23 (2.18 – 2.29) |
| Colorado | 6,394 | 408,571 | Yes | 2.12 (2.07 – 2.16) |
| Connecticut | 3,573 | 129,497 | Yes | 2.21 (2.15 – 2.26) |
| Delaware | 2,333 | 35,725 | Yes | 2.33 (2.24 - 2.41) |
| District of Columbia | 1,263 | 11,966 | Yes | 2.04 (1.95 – 2.14) |
| Florida | 16,725 | 687,220 | No | 2.37 (2.32 – 2.42) |
| Georgia | 3,228 | 336,273 | No | 2.44 (2.36 – 2.52) |
| Hawaii | 1,284 | 25,311 | Yes | 1.90 (1.82 – 1.98) |
| Idaho | 2,980 | 141,934 | No | 2.22 (2.14 – 2.30) |
| Illinois | 2,644 | 706,172 | Yes | 2.21 (2.13 – 2.29) |
| Indiana | 5,343 | 377,901 | No | 2.45 (2.39 – 2.51) |
| Iowa | 4,612 | 284,064 | Yes | 2.26 (2.20 – 2.33) |
| Kansas | 11,965 | 196,987 | No | 2.24 (2.21 – 2.27) |
| Kentucky | 6,161 | 242,384 | Yes | 2.52 (2.47 – 2.58) |
| Louisiana | 2,293 | 216,360 | No | 2.37 (2.24 – 2.58) |
| Maine | 4,618 | 119,700 | No | 2.39 (2.33 – 2.45) |
| Maryland | 5,564 | 226,978 | Yes | 2.27 (2.21 – 2.33) |
| Massachusetts | 7,213 | 272,807 | Yes | 2.26 (2.21 – 2.30) |
| Michigan | 6,189 | 559,120 | Yes | 2.43 (2.38 – 2.48) |
| Minnesota | 7,327 | 361,117 | Yes | 2.10 (2.06 – 2.14) |
| Mississippi | 2,925 | 162,459 | No | 2.49 (2.40 – 2.58) |
| Missouri | 3,517 | 499,909 | No | 2.34 (2.27 – 2.42) |
| Montana | 4,699 | 88,398 | No | 2.17 (2.11 – 2.22) |
| Nebraska | 9,357 | 179,029 | No | 2.18 (2.13 – 2.23) |
| Nevada | 2,192 | 121,494 | Yes | 2.27 (2.16 – 2.38) |
| New Hampshire | 3,576 | 84,199 | Yes | 2.32 (2.27 – 2.38) |
| New Jersey | 5,224 | 304,636 | Yes | 2.13 (2.08 – 2.18) |
| New Mexico | 2,784 | 90,490 | Yes | 2.25 (2.17 – 2.32) |
| New York | 3,533 | 680,871 | Yes | 2.22 (2.16 – 2.28) |
| North Carolina | 3,673 | 552,417 | No | 2.47 (2.40 – 2.54) |
| North Dakota | 4,032 | 54,878 | Yes | 2.14 (2.08 – 2.20) |
| Ohio | 6,200 | 599,773 | Yes | 2.41 (2.35 – 2.46) |
| Oklahoma | 3,694 | 260,533 | No | 2.44 (2.37 – 2.51) |
| Oregon | 3,038 | 199,040 | Yes | 2.41 (2.34 – 2.47) |
| Pennsylvania | 5,641 | 619,394 | Yes | 2.31 (2.26 – 2.36) |
| Rhode Island | 3,341 | 53,283 | Yes | 2.44 (2.38 – 2.50) |
| South Carolina | 4,146 | 287,006 | No | 2.39 (2.33 – 2.46) |
| South Dakota | 3,313 | 57,656 | No | 2.09 (2.02 – 2.16) |
| Tennessee | 3,015 | 289,849 | No | 2.54 (2.46 – 2.63) |
| Texas | 3,781 | 1,061,729 | No | 2.37 (2.30 – 2.45) |
| Utah | 6,357 | 220,596 | No | 2.29 (2.25 – 2.34) |
| Vermont | 3,563 | 51,593 | Yes | 2.26 (2.20 – 2.31) |
| Virginia | 3,686 | 351,527 | No | 2.36 (2.30 – 2.43) |
| Washington | 5,498 | 266,236 | Yes | 2.37 (2.32 – 2.42) |
| West Virginia | 3,254 | 212,069 | Yes | 2.64 (2.58 – 2.71) |
| Wisconsin | 3,145 | 459,549 | No | 2.18 (2.11 – 2.25) |
| Wyoming | 3,557 | 35,315 | No | 2.20 (2.11 – 2.28) |
| Total | 225,457 | 14,755,566 | - | 2.33 (2.32 – 2.34) |

| **Table 3: State-level mean number of comorbidities and Medicaid expansion status based on 2013 BRFSS.** Among White men alone. | | | | |
| --- | --- | --- | --- | --- |
| State | N | Estimated N | Expanded | Age-Adjusted Mean Comorbidities |
| Alabama | 1,620 | 145,750 | No | 2.45 (2.34 – 2.55) |
| Alaska | 1,596 | 48,208 | No | 1.94 (1.85 – 2.03) |
| Arizona | 1,305 | 225,584 | Yes | 2.15 (2.03 – 2.28) |
| Arkansas | 1,583 | 137,522 | Yes | 2.34 (2.23 – 2.45) |
| California | 2,926 | 893,043 | Yes | 2.04 (1.99 – 2.10) |
| Colorado | 4,846 | 372,137 | Yes | 1.99 (1.95 – 2.04) |
| Connecticut | 2,546 | 101,706 | Yes | 2.14 (2.08 – 2.20) |
| Delaware | 1,561 | 29,011 | Yes | 2.18 (2.09 – 2.27) |
| District of Columbia | 1,021 | 12,100 | Yes | 2.02 (1.91 – 2.12) |
| Florida | 10,643 | 587,531 | No | 2.28 (2.22 – 2.33) |
| Georgia | 2,067 | 287,009 | No | 2.27 (2.19 – 2.35) |
| Hawaii | 1,168 | 27,042 | Yes | 1.83 (1.73 – 1.92) |
| Idaho | 2,169 | 125,600 | No | 2.04 (1.96 – 2.13) |
| Illinois | 1,910 | 624,934 | Yes | 2.08 (1.99 – 2.17) |
| Indiana | 3,785 | 345,192 | No | 2.22 (2.16 – 2.29) |
| Iowa | 3,071 | 239,671 | Yes | 2.08 (2.01 – 2.15) |
| Kansas | 8,297 | 159,805 | No | 2.11 (2.08 – 2.15) |
| Kentucky | 3,403 | 177,426 | Yes | 2.35 (2.29 – 2.42) |
| Louisiana | 1,292 | 185,358 | No | 2.21 (2.07 – 2.34) |
| Maine | 3,176 | 97,253 | No | 2.21 (2.15 – 2.28) |
| Maryland | 3,656 | 179,242 | Yes | 2.11 (2.05 – 2.18) |
| Massachusetts | 4,775 | 226,940 | Yes | 2.16 (2.11 – 2.21) |
| Michigan | 4,665 | 485,351 | Yes | 2.33 (2.28 – 2.39) |
| Minnesota | 5,474 | 308,728 | Yes | 1.95 (1.91 – 1.99) |
| Mississippi | 1,772 | 137,581 | No | 2.34 (2.23 – 2.44) |
| Missouri | 2,276 | 384,701 | No | 2.26 (2.18 – 2.34) |
| Montana | 3,808 | 88,824 | No | 2.06 (2.00 – 2.11) |
| Nebraska | 6,419 | 150,013 | No | 2.08 (2.03 – 2.13) |
| Nevada | 1,630 | 104,460 | Yes | 2.11 (1.99 – 2.23) |
| New Hampshire | 2,549 | 66,728 | Yes | 2.24 (2.18 – 2.31) |
| New Jersey | 3,947 | 272,103 | Yes | 2.10 (2.05 – 2.16) |
| New Mexico | 2,094 | 80,295 | Yes | 2.12 (2.05 – 2.20) |
| New York | 2,621 | 640,307 | Yes | 2.14 (2.07 – 2.20) |
| North Carolina | 2,384 | 456,546 | No | 2.22 (2.14 – 2.29) |
| North Dakota | 3,310 | 57,769 | Yes | 2.03 (1.97 – 2.10) |
| Ohio | 4,191 | 479,973 | Yes | 2.21 (2.16 – 2.27) |
| Oklahoma | 2,413 | 207,975 | No | 2.27 (2.19 – 2.34) |
| Oregon | 2,217 | 169,411 | Yes | 2.20 (2.13 – 2.27) |
| Pennsylvania | 4,156 | 567,052 | Yes | 2.18 (2.13 – 2.23) |
| Rhode Island | 2,136 | 38,133 | Yes | 2.31 (2.24 – 2.38) |
| South Carolina | 3,099 | 256,192 | No | 2.33 (2.27 – 2.39) |
| South Dakota | 2,618 | 52,810 | No | 2.03 (1.95 – 2.12) |
| Tennessee | 1,810 | 213,296 | No | 2.27 (2.18 – 2.36) |
| Texas | 2,602 | 1,014,694 | No | 2.16 (2.08 – 2.23) |
| Utah | 5,085 | 219,140 | No | 2.05 (2.01 – 2.10) |
| Vermont | 2,530 | 38,790 | Yes | 2.16 (2.10 – 2.22) |
| Virginia | 2,744 | 316,413 | No | 2.12 (2.06 – 2.18) |
| Washington | 3,993 | 219,731 | Yes | 2.15 (2.10 – 2.21) |
| West Virginia | 2,298 | 156,709 | Yes | 2.49 (2.42 – 2.56) |
| Wisconsin | 2,523 | 415,991 | No | 2.10 (2.03 – 2.17) |
| Wyoming | 2,362 | 30,610 | No | 2.08 (2.00 – 2.17) |
| Total | 158,142 | 12,858,388 | - | 2.17 (2.16 – 2.18) |

| **Table 4: State-level mean number of comorbidities and Medicaid expansion status based on 2013 BRFSS.** Among Black women alone. | | | | |
| --- | --- | --- | --- | --- |
| State | N | Estimated N | Expanded | Age-Adjusted Mean Comorbidities |
| Alabama | 1,102 | 70,286 | No | 2.57 (2.44 – 2.70) |
| Alaska | 40 | 2,006 | No | 2.14 (1.52 – 2.76) |
| Arizona | 44 | 7,173 | Yes | 2.43 (1.71 – 3.15) |
| Arkansas | 518 | 34,923 | Yes | 2.55 (2.35 – 2.75) |
| California | 297 | 87,776 | Yes | 2.48 (2.27 – 2.69) |
| Colorado | 155 | 15,385 | Yes | 2.40 (2.10 – 2.71) |
| Connecticut | 423 | 13,925 | Yes | 2.44 (2.25 – 2.62) |
| Delaware | 488 | 10,049 | Yes | 2.62 (2.45 – 2.79) |
| District of Columbia | 1,467 | 14,937 | Yes | 2.57 (2.43 – 2.70) |
| Florida | 1,940 | 134,226 | No | 2.40 (2.26 – 2.53) |
| Georgia | 1,410 | 156,953 | No | 2.46 (2.35 – 2.57) |
| Hawaii | 14 | 455 | Yes | 2.20 (1.58 – 2.82) |
| Idaho | 5 | 174 | No | 2.42 (1.71 – 3.13) |
| Illinois | 314 | 122,700 | Yes | 2.47 (2.21 – 2.73) |
| Indiana | 385 | 37,344 | No | 2.47 (2.29 – 2.65) |
| Iowa | 64 | 3,908 | Yes | 2.50 (2.03 – 2.97) |
| Kansas | 574 | 9,120 | No | 2.48 (2.32 – 2.64) |
| Kentucky | 721 | 16,939 | Yes | 2.63 (2.40 – 2.86) |
| Louisiana | 1,033 | 105,782 | No | 2.52 (2.34 – 2.69) |
| Maine | 18 | 855 | No | 1.94 (1.33 – 2.54) |
| Maryland | 1,775 | 97,782 | Yes | 2.38 (2.28 – 2.47) |
| Massachusetts | 575 | 16,189 | Yes | 2.32 (2.11 – 2.52) |
| Michigan | 675 | 92,649 | Yes | 2.63 (2.50 – 2.77) |
| Minnesota | 327 | 13,389 | Yes | 2.41 (2.17 – 2.65) |
| Mississippi | 1,801 | 104,141 | No | 2.54 (2.43 – 2.66) |
| Missouri | 631 | 49,350 | No | 2.48 (2.25 – 2.70) |
| Montana | 8 | 158 | No | 4.28 (2.06 – 6.50) |
| Nebraska | 152 | 5,927 | No | 2.49 (2.16 – 2.82) |
| Nevada | 105 | 13,646 | Yes | 2.55 (2.10 – 2.99) |
| New Hampshire | 17 | 514 | Yes | 2.27 (1.77 – 2.77) |
| New Jersey | 1,071 | 72,199 | Yes | 2.41 (2.29 – 2.52) |
| New Mexico | 52 | 2,518 | Yes | 2.45 (1.92 – 2.98) |
| New York | 681 | 135,095 | Yes | 2.31 (2.16 – 2.45) |
| North Carolina | 1,093 | 194,181 | No | 2.59 (2.47 – 2.71) |
| North Dakota | 18 | 322 | Yes | 1.77 (0.57 – 2.97) |
| Ohio | 618 | 80,232 | Yes | 2.61 (2.47 – 2.76) |
| Oklahoma | 344 | 26,458 | No | 2.48 (2.25 – 2.72) |
| Oregon | 16 | 1,400 | Yes | 1.97 (1.52 – 2.43) |
| Pennsylvania | 631 | 75,775 | Yes | 2.64 (2.49 – 2.79) |
| Rhode Island | 165 | 3,105 | Yes | 2.59 (2.34 – 2.84) |
| South Carolina | 1,944 | 114,334 | No | 2.53 (2.44 – 2.63) |
| South Dakota | 10 | 333 | No | 1.54 (1.14 – 1.94) |
| Tennessee | 581 | 64,693 | No | 2.46 (2.30 – 2.62) |
| Texas | 526 | 195,599 | No | 2.61 (2.42 – 2.79) |
| Utah | 26 | 1,346 | No | 2.74 (1.65 – 3.83) |
| Vermont | 9 | 178 | Yes | 3.21 (2.53 – 3.90) |
| Virginia | 819 | 78,674 | No | 2.46 (2.34 – 2.59) |
| Washington | 115 | 8,367 | Yes | 2.49 (2.17 – 2.81) |
| West Virginia | 72 | 5,494 | Yes | 2.84 (2.41 – 3.27) |
| Wisconsin | 266 | 24,966 | No | 2.99 (2.58 – 3.39) |
| Wyoming | 16 | 146 | No | 2.92 (2.56 – 3.27) |
| Total | 26,151 | 2,324,074 | - | 2.51 (2.47 – 2.54) |

| **Table 5: State-level mean number of comorbidities and Medicaid expansion status based on 2013 BRFSS.** Among Black men alone. | | | | |
| --- | --- | --- | --- | --- |
| State | N | Estimated N | Expanded | Age-Adjusted Mean Comorbidities |
| Alabama | 488 | 45,950 | No | 2.29 (2.12 – 2.46) |
| Alaska | 28 | 1,112 | No | 2.24 (1.50 – 2.97) |
| Arizona | 51 | 15,058 | Yes | 2.15 (1.78 – 2.53) |
| Arkansas | 258 | 20,576 | Yes | 2.56 (2.27 – 2.85) |
| California | 226 | 77,026 | Yes | 2.35 (2.09 – 2.62) |
| Colorado | 137 | 17,663 | Yes | 2.13 (1.89 – 2.38) |
| Connecticut | 232 | 10,342 | Yes | 2.19 (1.97 – 2.40) |
| Delaware | 289 | 7,239 | Yes | 2.20 (2.00 – 2.40) |
| District of Columbia | 754 | 10,673 | Yes | 2.19 (2.04 – 2.35) |
| Florida | 1,007 | 92,246 | No | 2.22 (2.07 – 2.37) |
| Georgia | 671 | 106,080 | No | 2.12 (2.00 – 2.25) |
| Hawaii | 34 | 1,127 | Yes | 2.44 (1.91 – 2.98) |
| Idaho | 13 | 1,325 | No | 1.83 (1.01 – 2.64) |
| Illinois | 157 | 82,153 | Yes | 2.14 (1.93 – 2.35) |
| Indiana | 215 | 26,732 | No | 2.23 (2.00 – 2.46) |
| Iowa | 45 | 4,925 | Yes | 2.01 (1.51 – 2.51) |
| Kansas | 363 | 7,353 | No | 2.29 (2.10 – 2.48) |
| Kentucky | 268 | 10,257 | Yes | 2.15 (1.87 – 2.42) |
| Louisiana | 380 | 59,708 | No | 2.21 (1.97 – 2.46) |
| Maine | 24 | 1,387 | No | 2.35 (1.58 – 3.12) |
| Maryland | 820 | 63,223 | Yes | 2.03 (1.92 – 2.14) |
| Massachusetts | 312 | 14,848 | Yes | 2.05 (1.84 – 2.27) |
| Michigan | 379 | 69,007 | Yes | 2.47 (2.30 – 2.64) |
| Minnesota | 198 | 11,221 | Yes | 2.06 (1.83 – 2.30) |
| Mississippi | 734 | 63,126 | No | 2.12 (1.97 – 2.27) |
| Missouri | 286 | 33,238 | No | 2.06 (1.82 – 2.31) |
| Montana | 6 | 197 | No | 2.46 (1.64 – 3.29) |
| Nebraska | 109 | 5,322 | No | 2.35 (1.87 – 2.82) |
| Nevada | 81 | 13,113 | Yes | 1.84 (1.42 – 2.26) |
| New Hampshire | 21 | 930 | Yes | 1.88 (1.39 – 2.37) |
| New Jersey | 525 | 45,721 | Yes | 2.11 (1.96 – 2.26) |
| New Mexico | 55 | 3,089 | Yes | 2.18 (1.67 – 2.70) |
| New York | 354 | 98,591 | Yes | 2.23 (2.07 – 2.39) |
| North Carolina | 580 | 132,291 | No | 2.43 (2.26 – 2.60) |
| North Dakota | 24 | 779 | Yes | 2.29 (1.80 – 2.78) |
| Ohio | 335 | 57,783 | Yes | 2.17 (2.00 – 2.35) |
| Oklahoma | 189 | 16,433 | No | 2.17 (1.92 – 2.43) |
| Oregon | 13 | 1,408 | Yes | 2.32 (1.45 – 3.19) |
| Pennsylvania | 346 | 53,427 | Yes | 2.38 (2.20 – 2.56) |
| Rhode Island | 83 | 1,611 | Yes | 2.35 (1.93 – 2.77) |
| South Carolina | 1,036 | 75,025 | No | 2.28 (2.16 – 2.40) |
| South Dakota | 24 | 535 | No | 1.80 (1.42 – 2.19) |
| Tennessee | 245 | 34,478 | No | 2.47 (2.24 – 2.71) |
| Texas | 296 | 163,080 | No | 2.43 (2.19 – 2.67) |
| Utah | 29 | 1,475 | No | 2.68 (2.06 – 3.29) |
| Vermont | 11 | 237 | Yes | 2.38 (1.86 – 2.90) |
| Virginia | 423 | 54,786 | No | 2.08 (1.92 – 2.24) |
| Washington | 103 | 8,323 | Yes | 2.24 (1.91 – 2.56) |
| West Virginia | 48 | 4,575 | Yes | 2.30 (1.92 – 2.68) |
| Wisconsin | 136 | 20,187 | No | 2.58 (2.16 – 3.01) |
| Wyoming | 13 | 182 | No | 2.54 (1.53 – 3.56) |
| Total | 13,454 | 1,647,172 | - | 2.25 (2.21 – 2.30) |

**Figure 1: State-level mean number of comorbidities and Medicaid expansion status based on 2013 BRFSS. Among Non-Hispanic White women alone.**

**Figure 2: State-level mean number of comorbidities and Medicaid expansion status based on 2013 BRFSS. Among Non-Hispanic White men alone.**

**Figure 3: State-level mean number of comorbidities and Medicaid expansion status based on 2013 BRFSS. Among Non-Hispanic Black women alone.**

**Figure 4: State-level mean number of comorbidities and Medicaid expansion status based on 2013 BRFSS. Among Non-Hispanic Black men alone.**
